# Generation of DKK1 transgenic Tibet minipigs by somatic cell nuclear transfer (SCNT)

**DOI:** 10.18632/oncotarget.20604

**Published:** 2017-09-01

**Authors:** Wei Liu, Li-Hong Wu, Min Yue, Bayaer Nashun, Hua Tang, Yan Chen, Bang-Zhu Chen, Jin Yuan, Dong Xiao, Wei-Wang Gu

**Affiliations:** ^1^ Institute of Comparative Medicine and Laboratory Animal Center, Southern Medical University, Guangzhou 510515, China; ^2^ Pearl Laboratory Animal Sci. and Tech. Co. Ltd., Dongguan 523808, China; ^3^ Guangdong Provincial Key Laboratory of Cancer Immunotherapy Research, Guangzhou Key Laboratory of Tumor Immunology Research, Cancer Research Institute, Southern Medical University, Guangzhou 510515, China

**Keywords:** Dickkopf-related protein 1 (DKK1), lentivirus-mediated gene transfer, somatic cell nuclear transfer (SCNT), Tibet minipigs, transgenic pigs

## Abstract

Hairless mice have been widely applied in skin-related researches, while hairless pigs will be a useful model for skin-related study and other biomedical researches. Dickkopf-related protein 1 (DKK1) is inhibitor of Wnt signaling pathway. Transgenic mice expressing DKK1 transgene under control of a human keratin 14 (K14) promoter display hairless phenotype, which encouraged us to generate transgenic minipigs expressing pig DKK1 transgene under control of K14 promoter and finally achieve hairless minipigs. To generate transgenic cloned pigs, we constructed the lentiviral expression vector pERKDZG which contains two independent expression cassettes, the transcription of Tibet minipig DKK1 and EGFP genes are driven by K14 promoter, while mRFP is regulated under the control of Ef-1α promoter. Prior to generating the transgenic pig, the functionality of pERKDZG construct was verified by fluorescence assay and via checking pDKK1 expression. Subsequently, lentiviruses harboring ERKDZG transgene infected porcine embryonic fibroblasts (PEFs), followed by sorting RFP-positive PEFs by flow cytometry to obtain the purified PEFs carrying ERKDZG, designated DKK1-PEFs as donor cells used for somatic cell nuclear transfer (SCNT). Finally, we obtained 3 DKK1 transgenic cloned pigs with skin-specific expression of pDKK1 and EGFP transgenes, but unfortunately, DKK1 transgenic cloned pigs don't display hairless phenotype as expected. Taken together, we achieve DKK1 transgenic cloned pigs with skin-specific expression of pDKK1 transgene which provide a pig model for exploring DKK1 gene functions in pigs.

## INTRODUCTION

At present, the minipigs are becoming the most commonly used large laboratory animals because the minipigs are small in body size, easy to operate, and share anatomical, physiological and biochemical similarities with humans. Because of the remarkable similarities between porcine and human skin structure, pigs are often considered as an ideal model for skin-related studies, including skin grafting [1], cosmetic identification [2], ultraviolet radiation [3], skin cancer [4], burns [5, 6], frostbite [7], skin aging [8] and etc. However, because of pig skin coated with shaggy hairs, shaving hair process is inevitable before skin test or surgery.

Commonly used small laboratory animals without hair include nude mice, SKH hairless mice and hairless guinea pigs, etc [9]. SKH hairless mice have been widely applied in skin-related researches (i.e., hair tonic effect, skin allergies, skin grafting treatment and ultraviolet radiation response, etc) [10]. Hairless animals can eliminate the need for hair removal procedures before experiment and surgery, and avoid the skin damage caused thereby.

Currently, Yucatan miniature pig, a world’s only hairless pig strain, is employed in skin studies [11]. At present, China has nurtured many miniature pig strains, including Wuzhishan miniature pigs, Guizhou miniature pigs, Bama miniature pigs, Banna miniature pigs and Tibetan minipigs, etc. However, Chinese scientists has not yet nurtured hairless miniature pig strains, which are urgently required for biomedical researches.

Wnt proteins are required for initiating hair follicle development [12]. Dickkopf 1 (DKK1), a potent and specific endogenous secreted inhibitor of Wnt action, functions by binding and inhibiting LRP coreceptors required for activation of canonical Wnt signaling and is diffusible *in vivo* [12–14], and is associated with aging-associated alopecia in mice [15] and human [16]. Transgenic mice ectopically expressing DKK1 transgene under control of a human keratin 14 (K14) promoter displayed an early and complete block in the development of skin appendages including all types of hair follicle, thereby resulting in hairless phenotype [12]. Mice expressing lower levels of the DKK1 transgene were viable and displayed sparse hair or patches of absent hair [12], and the development of vibrissa follicles and all types of hair follicle is completely blocked by high levels of ectopic DKK1 in K14-Dkk1 transgenic mice [12]. Additionally, transgenic pigs harboring GFP transgene under control of a human K14 promoter showed GFP expression in the skin, but not in other organs [17]. Against this background, we intend to generate the transgenic minipigs expressing pig DKK1 (pDKK1) transgene under control of a human K14 promoter by somatic cell nuclear transfer (SCNT), and finally achieve hairless minipigs.

## RESULTS

### Construction of the lentiviral expression vector pERKDZG

**Figure 1:**
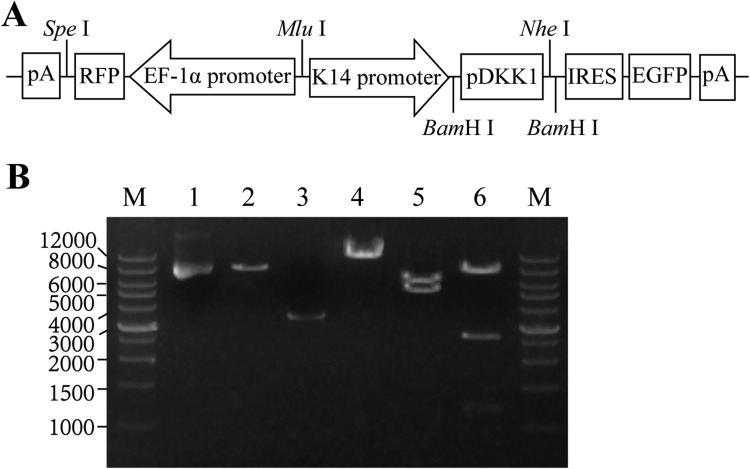
Construction of the lentiviral expression vector pERKDZG (**A**) Schematic illustration of the pERKDZG vector used to generate ERKDZG transgenic cloned pigs. (**B**) Identification of pERKDZG by enzyme digestion. Lane M: Wide Range DNA Marker (500–12,000) (TaKaRa); Lane 1: pERKDZG; Lane 2: Vector DNA; Lane 3: Insert DNA; Lane 4: pERKDZG cut by *Sal*I; Lane 5: pERKDZG cut by *Pvu*I; Lane 6: pERKDZG digested by *Nco*I. Abbreviations: EF-1α promoter: elongation factor 1*α* promoter; K14 promoter: human keratin 14 promoter; RFP: red fluorescent protein; EGFP: enhanced green fluorescent protein; pDKK1: pig DKK1 cDNA; IRES: internal ribozyme entry site; pA: polyadenylation signal.

pERKDZG was constructed via multiple steps of cloning as described in Methods, and the map is shown in Figure 1A. pERKDZG contains two independent expression cassettes, the transcription of pDKK1 and EGFP genes are driven by a human K14 promoter, while mRFP is regulated under the control of EF-1α promoter. The successful construction of pERKDZG was confirmed by restriction enzyme (Figure 1B) and sequencing (data not shown).

To express pDKK1 and EGFP transgenes in the porcine skin, we chose the human K14 gene promoter (Figure 1A) because it has been demonstrated to be strongly active in the dividing cells of the epidermal basal layer (basal keratinocytes), hair follicles and oral epithelia [18, 19]. Most importantly, the K14 promoter has been employed to achieve skin-specific expression of various transgene(s) in the transgenic mice [18, 19] and transgenic pigs [17]. As mentioned above, K14-GFP transgenic pig expressed GFP specifically in basal keratinocytes of skin [17]. Moreover, the skin-specific expression of pDKK1 in transgenic pig avoids the harmful effects of DKK1 overexpression in other tissues and organs.

The mRFP reporter gene (under the control of EF-1α promoter) contained in pERKDZG provides the following advantages. Firstly, mRFP is employed to assess whether the ERKDZG transgene is successfully introduced into PEFs by RFP assay. Secondly, mRFP-positive PEFs used as donor cells for SCNT can be sorted by FACS. Finally, the transgenic pigs are identified by RFP expression observation.

### *In vitro* confirming the functionality of pERKDZG

Prior to generating the transgenic pig, the functionality of pERKDZG construct was verified by fluorescence observation and via checking pDKK1 expression as described in “Materials and Methods” section.

**Figure 2:**
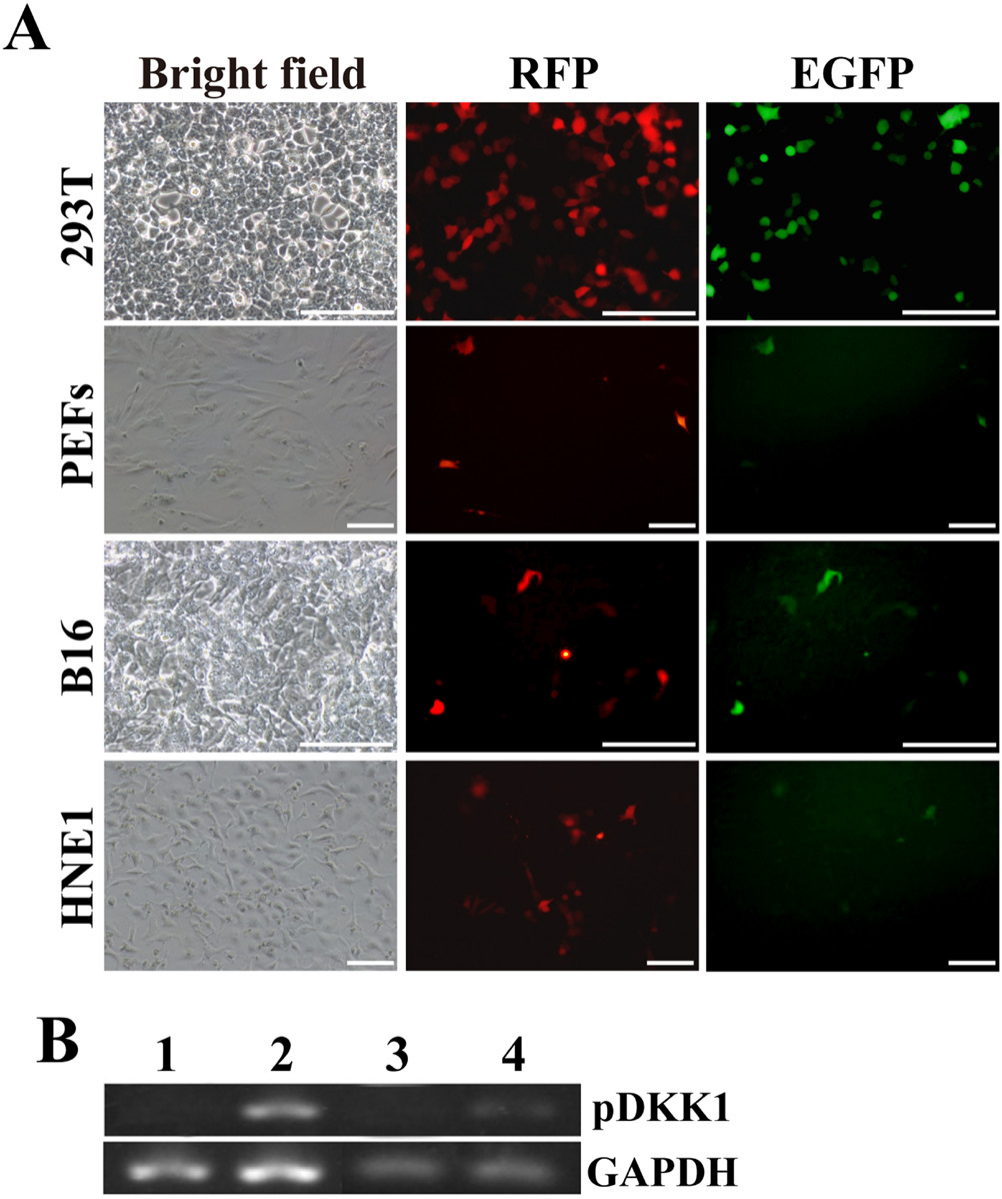
In vitro confirming the functionality of the resulting plasmid of pERKDZG (**A**) EGFP and RFP expression in PEFs, and 293T, B16 and HNE1 cells transiently transfected with pERKDZG. Scale bars: 200 μm. (**B**) RT-PCR detection of pDKK1 transgene expression in 293T cells and PEFs transiently transfected with pERKDZG. Lane 1: 293T cells transfected with pHEZG; Lane 2: 293T cells transfected with pERKDZG; Lane 3: PEFs transfected with pHEZG; Lane 4: PEFs transfected with pERKDZG.

As expected, RFP fluorescence was observed in the indicated cells transiently transfected with pERKDZG (Figure 2A), indicating that mRFP reporter gene can express normally under the control of a ubiquitous EF-1α promoter.

To *in vitro* examine the tissue or cell-type specificity of a K14 promoter contained in pERKDZG, four cell lines (i.e., 293T cells, PEFs, B16 cells and HNE1 cells) from different sources were transiently transfected with pERKDZG (Figure 2A). As expected, GFP fluorescence was detected under inverted fluorescence microscope in B16 cells (skin origin) transfected with pERKDZG (Figure 2A), whereas some 293T cells, PEFs and HNE1 cells transfected with pERKDZG unexpectedly displayed GFP fluorescence (Figure 2A). Additionally, nearly all of RFP-positive B16 cells transfected with pERKDZG showed strong green fluorescence, while a small number of RFP-positive cells (i.e., 293T cells, PEFs and HNE1 cells transfected with pERKDZG) exhibited weak GFP fluorescence (Figure 2A). The above-mentioned results prompted us to test the activity of K14 promoter in 293T cells, PEFs and HNE1 cells. Our results revealed that EGFP expression under control of K14 promoter was detectable in all of different type of cells (including 293T cells, PEFs, B16 cells and HNE1 cells) transiently transfected with pK14-GFP (Supplementary Figure 1), suggesting that human K14 promoter is active in these aforementioned cells. Moreover, RT-PCR analysis revealed that pDKK1 transgene expression was detected in 293T cells and PEFs transiently transfected with pERKDZG (Figure 2B). Together, these results confirm that the two independent expression cassette contained in the resultant plasmid of pERKDZG can normally work *in vitro*, which laid a solid foundation to produce DKK1 transgenic cloned pigs.

### Generation of transgenic donor cells used for SCNT

**Figure 3:**
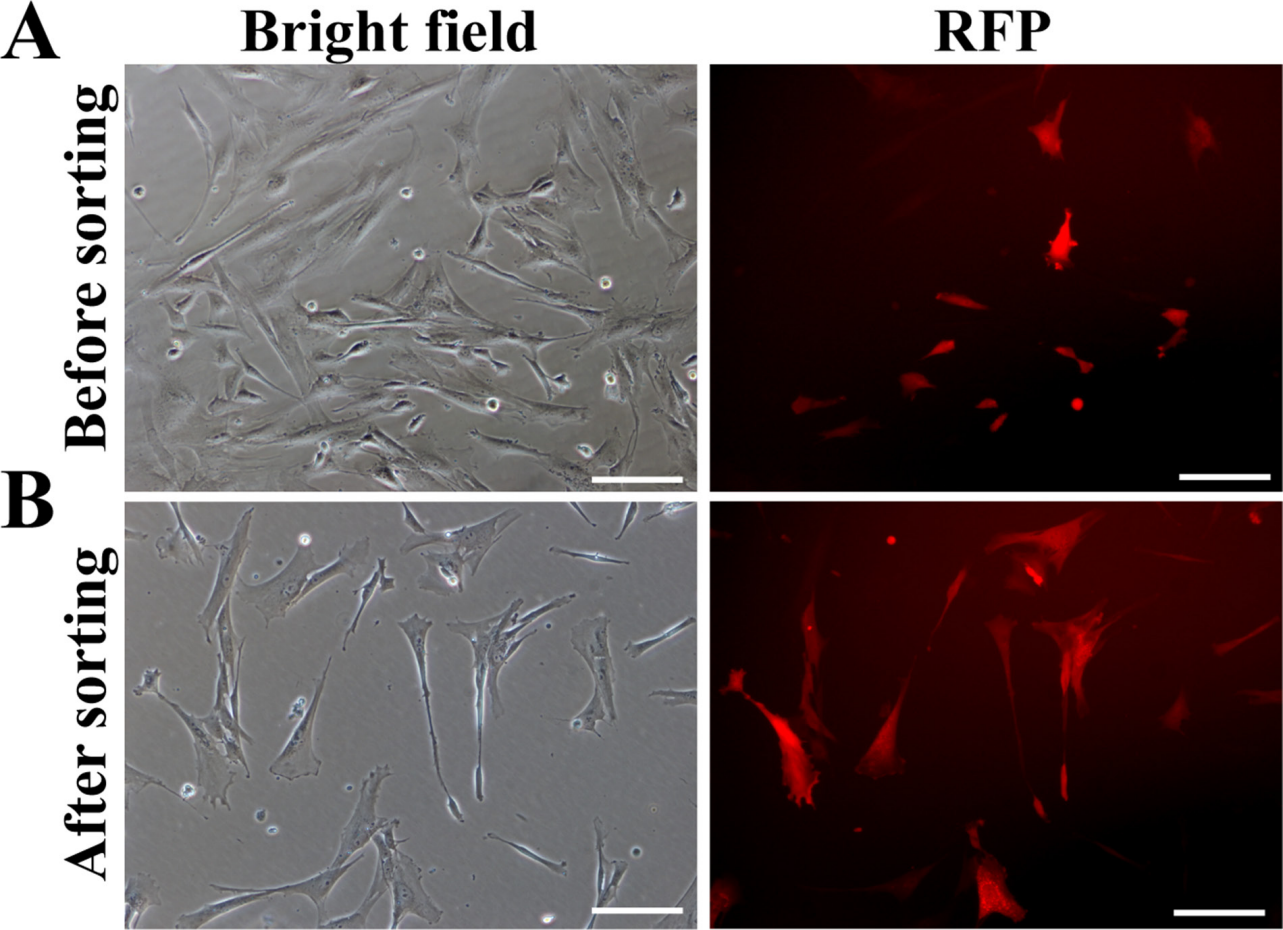
Generation of transgenic donor cells (**A**) PEFs infected by lentivirus harboring ERKDZG transgene 3 days after infection. Scale bars: 200 μm. (**B**) PEFs carrying ERKDZG transgene (designated PEF-DKK1) sorted by flow cytometry. Scale bars: 200 μm.

After the construct (e.g. pERKDZG) was confirmed for functionality, the lentiviruses harboring ERKDZG transgene were produced, followed by infecting PEFs. RFP expression illustrated that PEFs were successfully infected by lentiviruses harboring ERKDZG (Figure 3A). RFP-positive PEFs were sorted by flow cytometry to obtain the purified PEFs carrying ERKDZG (Figure 3B), designated DKK1-PEFs as donor cells used for SCNT.

### Generation of DKK1 transgenic pigs and genotype identification

**Figure 4:**
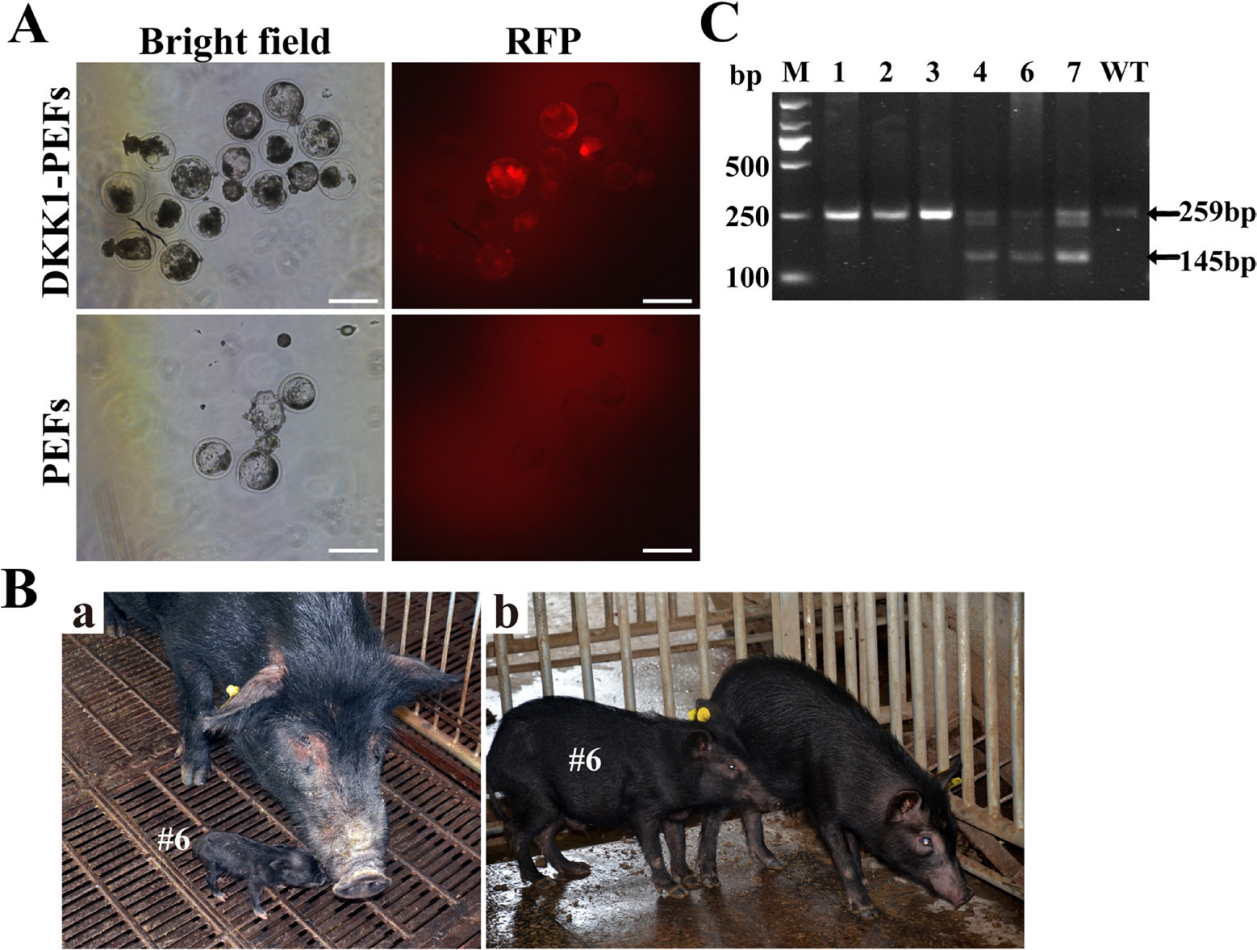
Generation of DKK1 transgenic cloned pigs by somatic cell nuclear transfer (SCNT) (**A**) RFP expression in transgenic reconstructed embryos. Scale bars: 200 μm. (**B**) DKK1 transgenic cloned pig #6. (**a**) 3-day-old DKK1 transgenic cloned pig #6; (**b**) 3-month-old DKK1 transgenic cloned pig #6. (**C**) PCR-based genotyping of DKK1 transgenic pigs. Lane M: DL2000 Marker; Lane 1–7: piglet #1, #2, #3, #4, #6, #7; WT: wild type piglet. (No samples were taken from Piglets #5 and #8 because they were lost on the following day after birth).

The transgenic donor cells (i.e., DKK1-PEFs) were employed to generate DKK1 transgenic Tibet minipigs by SCNT as described previously [20, 21]. The rate of *in vitro* blastocyst formation of reconstructed embryos is about 20% (data not shown). After *in vitro* culture for 7 days, the reconstructed embryos developed well and normally expressed RFP (Figure 4A), indicating that the exogenous transgene(s) can normally express in reconstructed embryos.

A total of 1088 reconstructed embryos were transferred to 7 recipient gilts that exhibited a natural estrus. Moreover, in order to obtain higher pregnancy rate, 110 reconstructed embryos derived from blank PEFs were mixed with 110 reconstructed embryos harboring ERKDZG transgene to be transferred to one recipient gilt (i.e., #110919). After gestation, 2 recipients were pregnant, and totally 8 cloned piglets were born by natural delivery. Cloned piglets #1–5 were born by recipient #110919, while cloned piglets #6–8 were born by recipient #101606 which 210 transgenic reconstructed embryos were transferred to. After genotype identification by PCR, piglets #4, #6 and #7 were identified as transgenic pigs (Figure 4C). Figure 4B-a and Figure 4B-b display the photos of 3-day-old DKK1 transgenic cloned pig #6 and 3-month-old DKK1 transgenic cloned pig #6, respectively.

### RFP, EGFP and pDKK1 transgenes expression in DKK1 transgenic pigs

**Figure 5:**
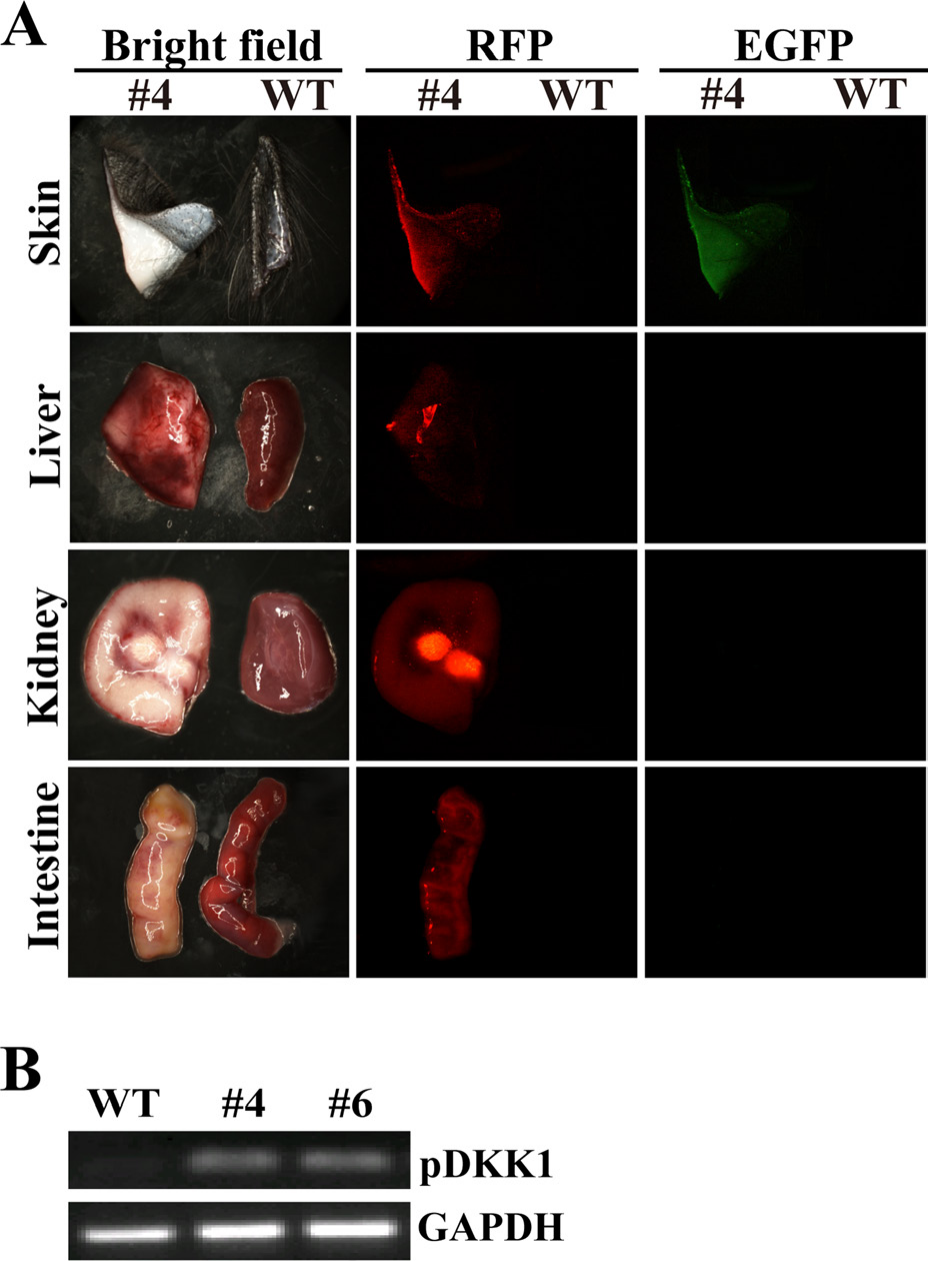
DKK1 transgene expression in DKK1 transgenic pigs (**A**) EGFP and RFP expression in different tissues of DKK1 transgenic pig #4. #4: organs from transgenic piglet #4; WT: organs from a wild type piglet; EGFP and RFP are detected under a stereo fluorescence microscope (Nikon, AZ100). (**B**) RT-PCR detection of pDKK1 transgene expression in skin of DKK1 transgenic pigs. WT: wild type piglet; #4 and #6: transgenic piglets #4 and #6.

We next determined the expression pattern of RFP and EGFP transgenes in organs and tissues taken from DKK1 transgenic cloned pigs. Red fluorescence was detected in tissue/organ samples including ear skin, muscle, heart, liver, spleen, lung, kidney, pancreas and intestine isolated from the transgenic pig, but not in control littermate (Figure 5A and Supplementary Figure 2), indicating ubiquitous expression of RFP transgenic cassette in DKK1 transgenic pigs. Green fluorescence was only detectable in ear skin, but not in muscle, heart, liver, spleen, lung, kidney, pancreas and intestine obtained from transgenic pig (Figure 5A and Supplementary Figure 2), suggesting skin-specific expression of EGFP transgene in DKK1 transgenic pigs. Moreover, pDKK1 transgene was detected in skin of DKK1 transgenic pigs (Figure 5B).

## DISCUSSION

As described in “Introduction section”, SKH hairless mice have been widely applied in skin-related researches [10]. Hairless pigs will be a useful model for skin-related study and other biomedical researches. Presently, Yucatan miniature pig, a world’s only hairless pig strain, is used in skin researches [11]. The important applications of hairless pigs encourage us to generate hairless miniature pig strains based on miniature pig strains nurtured by Chinese scientists in China. Transgenic mice expressing DKK1 transgene under control of a human K14 promoter displayed hairless phenotype [12]. Thus, we want to produce the transgenic minipigs expressing pDKK1 transgene under control of K14 promoter, and finally achieve hairless minipigs. In the present study, using SCNT, we have successfully generated transgenic cloned pigs that express pDKK1 transgene under control of K14 promoter, but none of DKK1 transgenic pigs showed hairless phenotype which was observed in K14-Dkk1 transgenic mice. The absence of hairless phenotype in DKK1 transgenic pigs may be due to the following possible reasons: 1) the expression level of pDKK1 transgene is not high enough to induce hairless phenotype, 2) the observing time is not enough and 3) DKK1 plays a different role in mice and pigs.

Wnt paracrine signaling molecules play key roles in the development of most organ systems, regulating cell fate decisions, proliferation, adhesion, cell shape and cell movements; the most extensively studied, ‘canonical’ pathway involves binding of Wnt to Frizzled (FZ) receptors and to obligate co-receptors of the LDL receptor related protein (LRP) family, leading to inactivation of a complex of proteins that degrades cytoplasmic b-catenin [22–24]. The previous studies revealed that Wnt signaling has emerged as the dominant pathway controlling the patterning of skin and influencing the decisions of embryonic and adult stem cells to adopt the various cell lineages of the skin and its appendages, as well as subsequently controlling the function of differentiated skin cells [22–24]. Additionally, the Wnt pathway is considered to be the master regulator during hair follicle morphogenesis in mice [22–24]. DKK1 is a specific endogenous secreted inhibitor of Wnt coreceptors of the LRP family that are required for the activation of the canonical Wnt signaling pathway [22–24]. Therefore, DKK1 transgenic pigs generated in this study may provide a large animal pig model for exploring the functions of Wnt signaling in skin development, homeostasis and disease, hair follicle development, and skin and hair follicle adult stem cells biology of pigs.

## MATERIALS AND METHODS

### Ethics statement

The animal experiment was approved by the Department of Science and Technology of Guangdong Province, with an approval ID SYXK (Guangdong) 2011–0074, and complied with the guidelines of the Animal Care Committee, Southern medical university.

### Vector construction

The plasmid pCDH-CMV-MCS-Ef1a-RFP was purchased from System Biosciences (SBI). The vector of pK14-mDKK1 was generously provided by Prof. Sarah E. Millar (University of Pennsylvania, Philadelphia, USA) [12]. The plasmid pHAGE-fullEf1a-MCS-IRES-ZsGreen (pHEZG) was a gift from Prof. Jeng-Shin Lee (Harvard Medical School, Boston, USA). The plasmid of pK14-GFP was generously provided by Prof. Alexander Pfeifer (University of Bonn, Bonn, Germany) [17].

Firstly, the full-length fragment of pig DKK1 (pDKK1) gene CDS was amplified by PCR from Tibet minipig liver cDNA with the primer pair (pDKK1-FP: 5′-GATGACGAAGAATGCGGCAG-3′, pDKK1-RP: 5′-CAAGGTGCTATGGTCATTA C-3′), which were designed based on reported pDKK1 complete CDS sequence (Genebank accession number: JN966758.1), and then inserted into pMD-18T simple (Takara) to generate pMD18-pDKK1, followed by sequencing. The sequencing results of two Tibet minipigs are consistent, and the CDS sequence of Tibet minipig DKK1 gene is shown in Supplementary Figure 3. Secondly, pDKK1 cDNA was amplified from pMD18-pDKK1, and subsequently subcloned into pK14-mDKK1 cutted by BamH I via In-Fusion cloning to generate pK14-pDKK1. Thirdly, a fragment of mRFP gene driven by EF-1α promoter (EF-1α-RFP) was amplified from pCDH-CMV-MCS-EF-1α-RFP, and then inserted into pHEZG at the Spe I site by In-Fusion cloning to creat pEf1α-RFP-fullEf1a-MCS-IRES-ZsGreen (pEREZG). Finally, the fragment of K14-pDKK1 was amplified from pK14-pDKK1, and subsequently subcloned into the MluI and NheI digested pEREZG by In-Fusion cloning to obtain the final transgenic vector pHAGE- EF-1α-RFP-K14-pDKK1-ZsGreen (pERKDZG) (Figure 1A). All of the above-mentioned vectors, including pK14-pDKK1, pEREZG and pERKDZG, were verified by sequencing (data not shown) and restriction enzyme digestion (Figure 1B or data not shown).

### Primary cell isolation and cell culture

Primary porcine embryonic fibroblasts (PEFs), were prepared as previously described [25–27]. Human embryonic kidney cell line 293T, human nasopharyngeal carcinoma cell line HNE1 and mouse skin melanoma cell line B16 were kept by our laboratory (Cancer Research Institute, Sourthern Medical University, Guangzhou, China). PEFs, 293T cells and B16 cells were maintained in DMEM medium supplemented with 10% fetal bovine serum (FBS, PAA), 1 mM glutamine (Gibco) and 1% nonessential amino acids (Gibco) in a humidified incubator with 5% CO2 at 37°C, while HNE1 cells were cultured in RPMI 1640 medium supplemented with 10% FBS in a humidified incubator with 5% CO2 at 37°C.

### *In vitro* identification of the functionality of the resulting pERKDZG

To *in vitro* confirm the functionality of the resulting plasmid pERKDZG, pERKDZG was transiently transfected into PEFs, 293T cells, B16 cells and HNE1 cells by lipofectamine 2000 reagent (Invitrogen) according to the manufacturer’s instructions, and then the expression of EGFP and RFP in transfected cells were assayed under inverted fluorescence microscope (Nikon TE-2000) 48h after transfection, and pDKK1 transgene expression was detected by RT-PCR.

### Generation of transgenic donor cells used for SCNT

The lentiviral packaging plasmids psPAX2 and pMD2.G were kindly provided by Prof Didier Trono (University of Geneva, Geneva, Switzerland). To generate transgenic donor cells, the lentiviral expressing vector pERKDZG along with packaging plasmids (psPAX2 and pMD2.G) were co-transfected into 293T cells using Lipofectamine 2000 reagent (Invitrogen) according to the manufacturers’ instruction, and subsequently virus supernatant was harvested 96 hours after transfection to infect PEFs. 3 days later, the infecting efficiency was estimated by RFP assay and FACS analysis. Next, RFP-positive PEFs were sorted by flow cytometry to obtain the purified PEFs carrying ERKDZG, designated PEF-DKK1 as donor cells used for SCNT.

### Generation of DKK1 transgenic pigs by SCNT

SCNT was performed as described [20, 21]. Pig oocytes were collected from ovaries purchased from a local slaughter house and cultured for 42–44 h for maturation. Then the nuclei of the *in vitro* matured oocytes were removed by micromanipulation. After electro-fusion and activation, about 200 reconstructed embryos were surgically transferred into the oviduct of a surrogate pig on the first day of standing estrus. Some embryos were cultured for 7 days to test the blastocyst formation rate as well as developmental ability. RFP expression in the blastocysts were observed under the inverted fluorescence microscope. Pregnancy status was monitored using an ultrasound scanner between 30–35 days post-transplantation and the cloned piglets were delivered by natural birth.

### Genotype analysis by PCR

Genomic DNA was extracted from the ears of newborn piglets. To identify whether the exotic DKK1 transgene was integrated into the genomic DNA, two primer pairs were designed and synthesized to amplify the genes DKK1 and GAPDH (used as the reference gene). The primers for DKK1 gene are forward pair (5′- GGGGAAATTGAGGAAACC-3′) and reverse pair (5′-CACAGTCTGATGATCGGAGA-3′), while the primers for GAPDH gene are forward pair (5′-TTGGCTACAGCAACAGGG-3′) and reverse pair (5′-CTGGGATGGAAACTGGAAGT-3′). PCR amplification conditions are as follows: pre-denaturationat 94°C for 5min, followed by 30 amplification cycles of denaturation at 94°C for 50 s, primer annealing at 60°C for 40s, and extension at 72°C for 30 s, and finally an additional extension at 72°C for 10min. Genomic DNA from a wild type piglet was used as a negative control. The PCR products were analyzed by 3% agarose gel electrophoresis.

As the forward and reverse primers for DKK1 gene are located in different exons, PCR products amplified from the genomic DNA is 259bp and PCR products amplified from the exogenous DKK1 transgene is 145bp. Based on the primer design for DKK1 gene, 259bp and 145 bp PCR products should be amplified from the genomic DNA of DKK1 transgene cloned pigs, and only 259 bp PCR products should be amplified from the genomic DNA of wildtype pigs.

### Organ fluorescence imaging

For organ (*ex vivo*) imaging, fresh organs and tissues (including ear skin, muscle, heart, liver, spleen, lung, kidney, pancreas and intestine) from transgenic pig #4 and wild-type littermate piglet were placed on 10cm plates and observed under the stereo fluorescent microscope (Nikon, AZ100).

### RT-PCR

Total RNA was extracted from cells transfected by pERKDZG and skin of DKK1 transgenic piglets using Trizol Reagent (TaKaRa), and then treated with DNase I (TaKaRa) according to the protocol provided by the manufacturer. First-strand cDNA was synthesized using the PrimeScript RT reagent Kit (TaKaRa). The mRNA expression levels of DKK1 transgene were determined by RT-PCR. The primers used for RT-PCR to detect the expression of DKK1 transgene and GAPDH gene (used as the reference gene) are the same as primers used for genotype identification (described above). PCR amplification conditions are the same as amplification conditions for PCR-based genotyping. The PCR products (145 bp for DKK1 and 186 bp for GAPDH) were then analyzed by 3% agarose gel electrophoresis.

## SUPPLEMENTARY MATERIALS FIGURES


