# Supplementary material for: Generation of DKK1 transgenic Tibet minipigs by somatic cell nuclear transfer (SCNT)
Source: Oncotarget. 2017 Sep 1;8(43):74331–9. doi: 10.18632/oncotarget.20604 (PMC5650344; doi:10.18632/oncotarget.20604)
Supplement: Supplementary file 1 [file oncotarget-08-74331-s001.pdf]

Generation of DKK1 transgenic Tibet minipigs by somatic cell nuclear transfer (SCNT)

SUPPLEMENTARY MATERIALS

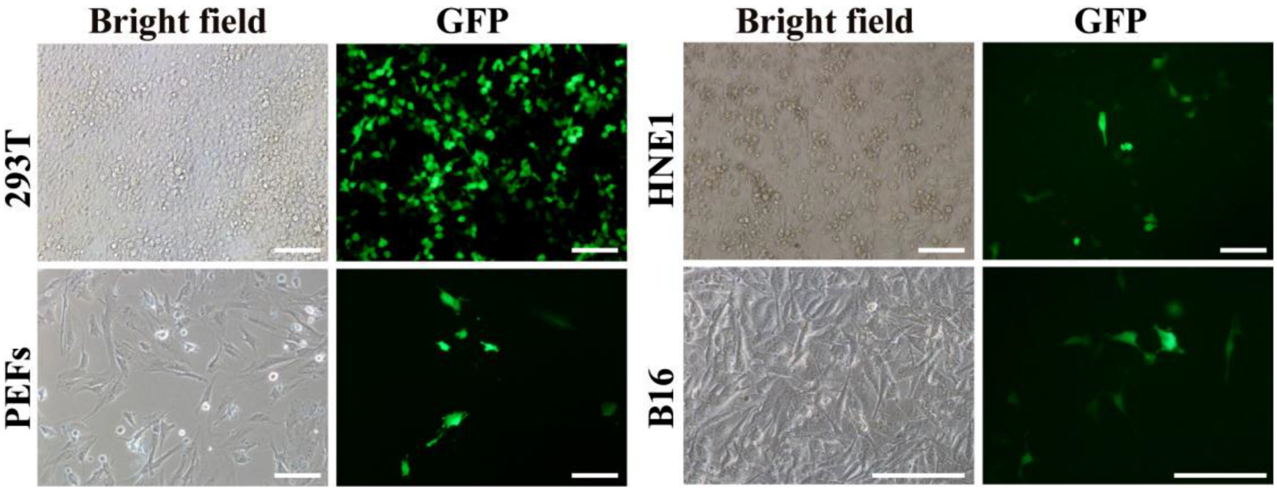

Supplementary Figure 1: GFP expression in different type of cells transfected with pK14-GFP. Scale bars: 200 μm.

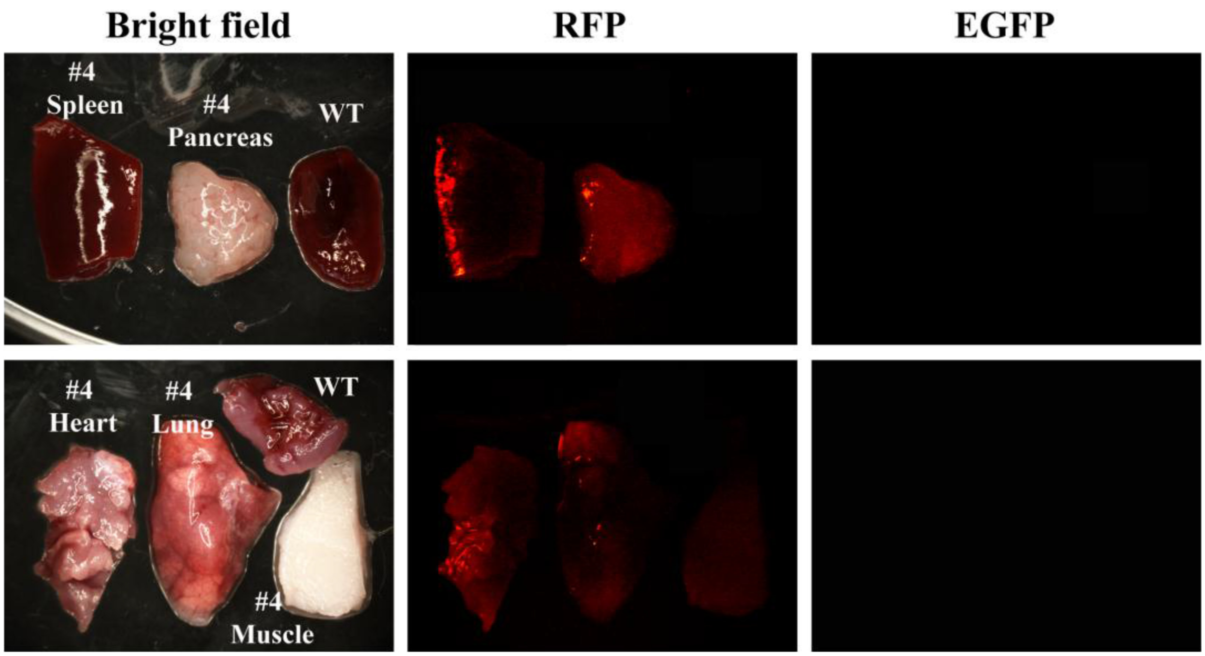

Supplementary Figure 2: EGFP and RFP expression in different tissues of DKK1 transgenic pig. #4: transgenic piglet #4; WT: organs from a wild-type piglet; EGFP and RFP were observed under a stereo fluorescence microscope.

5′-ATGAGGGCTCTGGCTGCAGCGGGTGCTGCCCGGGTCTTGGTCACCCTGGCAGCTGCGGCTCTTTGCGGTCAC

CCTCTGCTGGGAGCGAGCGCCACTTTAAACTCGGTTCTCGTCAATTCCAACGCCATCAAGAACCTGCCCCCACCGC

TGGGCGGCGCTGCGGGGCACCCGGCAGTCAGCGCGGCTCCCGGAATTCTGTTCGAGGGTGGCAACAAGTACCAA

ACGATTGACAACTATCAGCCATACCCGTGCGCCGATGACGAAGAATGCRGCAGTGACGAGTACTGCGCGAGTCCC

ACCCGCGGAGGAAGTGCAGGCGCACAAATCTGCCTAGCCTGCAGAAAGCGCCGAAAACGCTGCATGCGGCACGC

TATGTGCTGCCCTGGGAATTACTGCAAAAACGGAATATGTATGCCTTCTGATCACAATCATTTCCACCGAGGGGAAA

TTGAGGAAACCATTATTGAAAGCTTTGGTAATGACCATAGCACCTTGGATGGGTACTCCAGAAGAACTACACTGTC

ATCAAAAATGTATCATACCAAAGGGCAAGAAGGTTCTGTCTGTCTCCGATCATCAGACTGTGCCACAGGGTTGTGT

TGTGCAAGACATTTCTGGTCCAAGATCTGTAAACCTGTCCTCAAAGAAGGTCAAGTATGCACCAAGCACAGGAGAA

AAGGTTCCCATGGGCTGGAGATATTCCAGCGTTGTTACTGTGGAGAAGGTCTGTCTTGCCGGATACAGAAAGATC

ACCATCAAGCCAGTAATTCTTCTAGACTTCACACCTGTCAGAGACACGATTACAAGGATGACGACGATAAGTAA-3′

Supplementary Figure 3: The coding sequence of Tibet minipig DKK1 gene.
